# *Schistosoma mansoni* infection suppresses the growth of *Plasmodium yoelii* parasites in the liver and reduces gametocyte infectivity to mosquitoes

**DOI:** 10.1371/journal.pntd.0006197

**Published:** 2018-01-26

**Authors:** Taeko Moriyasu, Risa Nakamura, Sharmina Deloer, Masachika Senba, Masato Kubo, Megumi Inoue, Richard Culleton, Shinjiro Hamano

**Affiliations:** 1 Leading Program, Graduate School of Biomedical Sciences, Nagasaki University, Nagasaki, Japan; 2 Department of Parasitology, Institute of Tropical Medicine (NEKKEN), Nagasaki University, Nagasaki, Japan; 3 The Joint Usage/Research Center on Tropical Disease, Institute of Tropical Medicine (NEKKEN), Nagasaki University, Nagasaki, Japan; 4 Pathology Unit, Department of Pathology, Institute of Tropical Medicine (NEKKEN), Nagasaki University, Nagasaki, Japan; 5 Laboratory for Cytokine Regulation, Research Center for Integrative Medical Science (IMS), RIKEN Yokohama Institute, Yokohama, Japan; 6 Division of Molecular Pathology, Research Institute for Biomedical Science, Tokyo University of Science, Noda, Japan; 7 Malaria Unit, Department of Pathology, Institute of Tropical Medicine (NEKKEN), Nagasaki University, Nagasaki, Japan; Queen's University Belfast, UNITED KINGDOM

## Abstract

Malaria and schistosomiasis are major parasitic diseases causing morbidity and mortality in the tropics. Epidemiological surveys have revealed coinfection rates of up to 30% among children in Sub-Saharan Africa. To investigate the impact of coinfection of these two parasites on disease epidemiology and pathology, we carried out coinfection studies using *Plasmodium yoelii* and *Schistosoma mansoni* in mice. Malaria parasite growth in the liver following sporozoite inoculation is significantly inhibited in mice infected with *S*. *mansoni*, so that when low numbers of sporozoites are inoculated, there is a large reduction in the percentage of mice that go on to develop blood stage malaria. Furthermore, gametocyte infectivity is much reduced in mice with *S*. *mansoni* infections. These results have profound implications for understanding the interactions between *Plasmodium* and *Schistosoma* species, and have implications for the control of malaria in schistosome endemic areas.

## Author summary

Malaria and schistosomiasis are parasitic infectious diseases that cause severe morbidity and mortality in the tropics. Chronic schistosomiasis causes malnutrition and impaired intellectual development to children while malaria can cause fatal acute infections. Since coinfection of these two parasites is common in the tropics, many studies of both epidemiology and coinfection in animal models have been performed in order to reveal interactions between them. Previous animal studies on the interactions between *Plasmodium* and *Schistosoma* parasites have focused on the blood stage pathology of the malaria infection, and have consistently shown that parasitaemia can be enhanced in the presence of the helminth. In contrast, we focused on liver immunopathology in mice during coinfection between with *Schistosoma* and *Plasmodium*. We show that *S*. *mansoni* infection inhibits *Plasmodium* parasite growth in the liver resulting in a large reduction in the percentage of mice that go on to develop blood stage malaria following inoculation of low numbers of sporozoites. We also demonstrate that gametocyte infectivity is much reduced in mice with *S*. *mansoni* infections. Our results imply that *S*. *mansoni* infection can reduce malaria transmission both from mosquitoes to mice, and from mice to mosquitoes.

## Introduction

Malaria and schistosomiasis are two of the most important parasitic diseases in the tropics, and together constitute a severe burden to public health and to the economic development of endemic countries. Malaria is estimated to cause 429,000 deaths per year, 70% of those occurring in children aged under five years old [1]. The WHO has estimated that schistosomiasis causes about 200,000 deaths every year in sub-Saharan Africa and 218 million people were required to undergo preventive chemotherapy against the helminth globally in 2015.

The ranges of *Plasmodium* and *Schistosoma* overlap in much of the tropical world, leading to the potential for a great many coinfections of the two parasitic species. It has, for example, been estimated that there may be a greater than 30% coinfection rate among children in Sub-Saharan Africa [2]. Given the importance of such coinfections, interactions between *Plasmodium* and *Schistosoma* have been extensively studied both in nature, and using animal models in the laboratory.

Epidemiological studies on coinfections have often produced contrasting results: some reports contend that *Schistosoma* infection can increase susceptibility to *Plasmodium falciparum* [3–5], whilst others document a protective effect on *P*. *falciparum* incidence [6–9]. Differences in study design, genetic background of host populations and other environmental factors presumably contribute to these conflicting results.

Most laboratory-based animal studies have shown an exacerbation of malaria parasitaemia in *Schistosoma* infected mice [10–13] whilst others have revealed a protective effect of *Schistosoma* infection against experimental cerebral malaria and associated mortality [14–17]. In experimental *S*. *mansoni* infections, it is known that eggs deposited in the liver induce a strong Th2 type immune response [18]. Previous work has suggested that the exacerbation of malaria parasitaemia and protection against experimental cerebral malaria were mediated by a polarized Th2 immune environment which down-modulates inflammatory responses [10, 15].

The interactions between *Schistosoma* and *Plasmodium* are mainly mediated via host immune responses [19, 20]. Previous animal studies have investigated inter-species interactions using experimental infection with *Schistosoma* cercariae and *Plasmodium*-parasitized erythrocytes. As both parasites infect the liver at specific stages in their life cycle, we have focused on the immune reactions against those parasites in the liver. The major immunopathology in schistosomiasis is induced by egg-derived antigens in the liver, and hepatic immune cells develop immunity not only against pre-erythrocytic stages but also the blood stages of malaria parasites [21–24]. We therefore investigated whether an ongoing infection with *S*. *mansoni* could affect the rodent malaria parasite *Plasmodium yoelii* in the livers of mice challenged with sporozoites (SPZ).

We also evaluated whether *S*. *mansoni* infection affects malaria parasite gametocyte infectivity to mosquitoes, as it has been shown that the infectivity of malaria gametocytes decreases during the early stage of malaria infection due to host serum-mediated immunity [25–27].

## Methods

### Ethical statement

All mouse experiments were approved by the Institutional Animal Research Committee of Nagasaki University (No.1506181240) and performed according to Japanese law for the Humane Treatment and Management of Animals (Law No. 105 dated 19 October 1973 modified on 2 June 2006).

### Mice

Six week-old female BALB/cCrSlc (hereafter referred to as BALB/c) and C57BL/6NCrSlc (hereafter referred to as B6) mice were purchased from Japan SLC, Inc. (Shizuoka, Japan). Six week-old female CBA/J mice were purchased from Charles River Laboratories Japan, Inc. (Kanagawa, Japan). IFN-γ-deficient (IFN-γ^-/-^) mice and IL-4-deficient (IL-4^-/-^) mice were produced at RIKEN Yokohama Institute, Yokohama, Japan. All mice were maintained in the animal facilities of Nagasaki University with environmentally controlled, specific pathogen free conditions. Experiments were conducted with BALB/c mice unless otherwise specified.

### Parasites and infections

The rodent malaria parasites *Plasmodium yoelii yoelii 17x1*.*1pp* [28] (hereafter referred to as *P*. *yoelii*) and *Plasmodium berghei ANKA (hereafter referred to as P*. *berghei)* were used throughout these experiments. A Puerto Rican strain of *Schistosoma mansoni* was maintained in the animal facilities of Nagasaki University by passage through *Biomphalaria glabrata* snails and ICR mice or *Meriones unguiculatus (*Mongolian jirds). In the coinfection model, experimental mice were percutaneously infected with 50 cercariae. In the intraportal infusion model, eggs were collected from the liver harvested from ICR mice after 7 weeks infection with 250 cercariae and stored at -30°C until use. The mice were anesthetized and inoculated with 3,000 eggs in a total volume of 100 μL PBS via the portal vein.

Infections with *P*. *yoelii* and *P*. *berghei* were performed by i.v. inoculation with SPZ collected from *Anopheles stephensi* mosquitoes, as previously described [29]. 50, 500, or 1,500 SPZ in a total volume of 100 μl PBS were inoculated for assessment of malaria parasitaemia or malaria parasite liver burden. Malaria challenges were performed 10 weeks after *S*. *mansoni*-cercariae infection in the coinfection model and 3 weeks after *S*. *mansoni* egg inoculation in the intraportal infusion model.

In transmission experiments, mice were intravenously infected with one million *P*. *yoelii*-parasitized erythrocytes. On day 3, 4, and 5 after *P*. *yoelii* infection, six-day-post emergence female *A*. *stephensi* mosquitoes were allowed to feed on individual mice for 30 minutes and reared in the insectary at 24°C with 80% humidity for seven days. Midguts were harvested from the mosquitoes and oocysts counted by light microscopy.

### Malaria parasite density in the blood and the liver

Ten microliters of blood were collected from the tale-vein of mice at each time point. Livers were harvested 42 hours after malaria challenge infection. DNA was extracted from blood or livers using the EZ1 BioRobot (Qiagen N.V., Hilden, Germany) following the manufacturer’s instructions. Real-time quantitative PCR (qPCR) was performed on DNA samples using the Applied Biosystems 7500 Real Time PCR system (Thermo Fisher Scientific, Inc., Massachusetts, USA). A master mix of the following reaction components was prepared: 4.75 μL water, 6.25 μL Power SYBR Green Master Mix (Qiagen N.V., Hilden, Germany), 0.25 μL forward primer (10 μM), and 0.25 μL reverse primer (10 μM). 5 μL DNA samples were added as PCR template. *P*. *yoelii* or *P*. *berghei* 18s gene was amplified using the primers 18s F1 5’ GGAACGATGTGTGTCTAACACAAGGA 3’ and 18s R1 CGCGTGCAGCCTAGTATATCTAAGGACA 3’ (Table 1). Copy numbers of the parasite 18s gene were quantified with reference to a standard curve calculated from known numbers of plasmids containing the same gene sequence, as previously described [30]. The copy number of parasite 18s gene in each sample was standardized by the simultaneous quantification of the mouse glyceraldehyde 3-phosphate dehydrogenase (G3PDH) gene. The mouse G3PDH gene was amplified using the primers MmG3PDHF1 5’ CATCTGAGGGCCCACTGAAG 3’ and MmG3PDHR1 5’ TGCTGTTGAAGTCGCAGGAG 3’(Table 1).

**Table 1:** Information for PCR primers.

Gene name	Accession No	Primer sequence	Product size
*Plasmodium* 18s	M14599.1	F: GGAACGATGTGTGTCTAACACAAGGA	80bp
		R: CGCGTGCAGCCTAGTATATCTAAGGACA	
Mouse G3PDH	NM_008084.3	F: CATCTGAGGGCCCACTGAAG	72bp
		R: TGCTGTTGAAGTCGCAGGAG	

### Monitoring of parasitaemia and gametocytaemia

Thin blood films from the tail-vein were prepared daily from day 2 to 8 after SPZ inoculation, and stained with Giemsa’s solution. The numbers of infected and non-infected erythrocytes ware counted per 10 microscopic fields to calculate parasitaemia and gametocytaemia.

### Flow cytometric analysis

Hepatic nonparenchymal cells were isolated after *Schistosoma* inoculation as follows. Livers taken from each experimental mouse were homogenized in 5 mL of culture medium (RPMI-1640 supplemented with 10% FCS and 1% penicillin/streptomycin) using gentleMACS Dissociator (Miltenyi Biotec, Bergisch Gladbach, Germany), filtered through a mesh and suspended in HBSS. To remove liver parenchymal cells, the cells were resuspended in 33% Percoll (GE Healthcare UK Ltd., Buckinghamshire, England) containing 2.5mL of 5000U/5mL Heparin (Mochida Pharmaceutical Co., Ltd., Tokyo, Japan) and were centrifuged at 900 g for 20 min at room temperature. The pellet was resuspended in RBC lysis buffer then washed in HBSS, and resuspended in the culture medium.

The following mAbs were used for flow cytometric analysis using BD FACSVerse (Nippon Becton Dickinson Company, Ltd., Tokyo, Japan): PE anti-mouse CD3e (145-2C11), and PE anti-mouse T-bet (eBio4B10) (Affymetrix, Inc., California, U.S.), PE-Cy7 anti-mouse CD 4 (GK1.5), Percp-Cy5.5 anti-mouseCD3e (145-2C11), PE-Cy7 anti-mouse F4/80 (BM8), APC anti-mouse Gata3 (16E10A23), APC anti-mouse TCRgd (GL3), PE anti-mouse Ly6G (Gr1)(RB6-8C5), FITC anti-mouse CD11b (M1/70), and Biotin anti-mouse F4/80 (BM8) (BioLegend, California, U.S.), FITC anti-mouse CD49b (DX5), and BV421-streptavidin (Becton, Dickinson and Company, New Jersey, U.S.).

Fixation, permeabilization, and staining of the target transcription factors (T-bet and GATA-3) were conducted with Foxp3/Transcription Factor Staining Buffer Set (Affymetrix Japan K.K., Tokyo, Japan) according to the manufacturer’s instructions.

### Data analysis

Data analyses were performed using Microsoft Excel 2010 (Microsoft Corporation, Washington, USA) and GraphPad Prism6 (GraphPad Software, Inc., California, USA). Significance between control and treatment groups was determined with Student’s t-tests. Survival and protection rates were statistically examined using the log-rank test. P values less than 0.05 were considered significant.

## Results

### *S*. *mansoni* infection significantly reduces the number of malaria parasites in the liver following sporozoite inoculation

To investigate the impact of chronic *S*. *mansoni* infection on the growth of malaria parasites in the liver, BALB/c mice were infected with 50 cercariae subcutaneously 10 weeks prior to i.v. inoculation of 1,500 *P*. *yoelii* sporozoites (SPZ). The number of copies of the malaria parasite 18s gene present in the liver of mice was measured by qPCR 42 h after SPZ challenge. Parasitaemia was monitored daily from day 2 to 8 post SPZ challenge.

**Fig 1:**
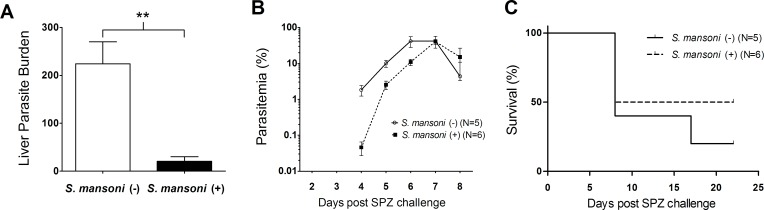
Growth of malaria parasites in the liver and blood of mice following SPZ inoculation of *Plasmodium yoelii* with and without *Schistosoma mansoni* infection. (A) Copy number of *P*. *yoelii* 18s RNA gene per 1×10^6^mouse G3PDH gene measured at 42 h post SPZ inoculation. Female BALB/c mice (N = 6) infected with 50 *S*. *mansoni*-cercariae 10 weeks previously were challenged with 1,500 *P*. *yoelii* SPZ along with *S*. *mansoni*-non-infected controls. **P<0.01, Student’s two-tailed t-test, t = 4.362, df = 10. (B) Parasitaemia. Blood stage malaria parasites were monitored daily from day 2 to 8 post i.v. inoculation of 500 *P*. *yoelii* SPZ. (C) Percentage survival. Data from one representative experiment of three independent repeats are shown.

Malaria parasite liver burden was significantly reduced to one-tenth or less in *S*. *mansoni*-infected mice compared with non-*S*. *mansoni* infected controls (Fig 1A). This reduction in the numbers of malaria parasites in the liver resulted in a delay in the onset of parasitaemia in the *S*. *mansoni*-infected group. All mice in both groups developed blood stage malaria parasite infection, and the peak parasitaemia was not affected by *S*. *mansoni* infection (Fig 1B). There was no difference in mortality between *S*. *mansoni*-infected and non-infected mice (Fig 1C).

### The reduction of liver-stage malaria parasite burden induced by *S*. *mansoni* infection was not constrained by malaria species or mouse strains

The genetic background of inbred mouse strains can determine the course of experimentally induced malaria parasite infections [31, 32]. We examined the malaria parasite liver burden in three different mouse strains; BALB/c, B6, and CBA/J in coinfections with *S*. *mansoni*. Female BALB/c, B6, and CBA/J mice were challenged with two different *Plasmodium* species: *P*. *yoelii* and *P*. *berghei* in order to investigate whether the reduction of malaria parasite liver burden occurs across mouse and parasite species.

**Fig 2:**
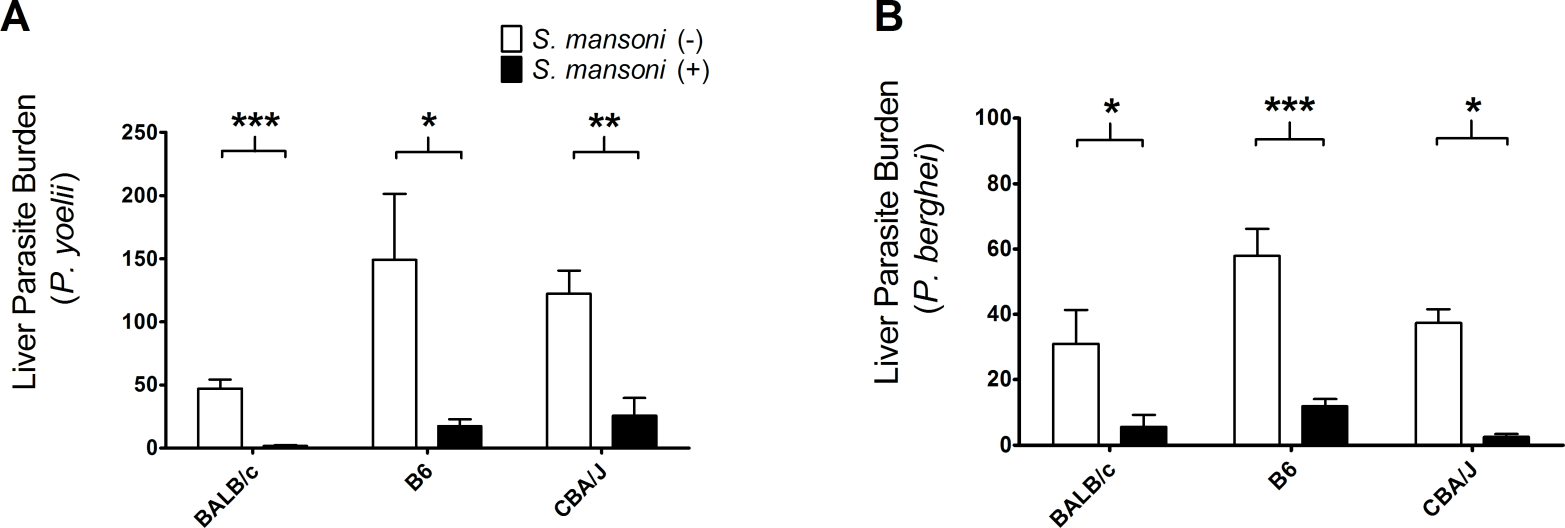
Malaria parasite liver burden in BALB/c, C57BL/6, and CBA/J mice. (A) Copy number of *Plasmodium yoelii* 18s RNA gene per 1×10^6^ mouse G3PDH gene measured at 42 h post sporozoite inoculation. Female BALB/c, B6, and CBA/J mice (N = 5) infected with 50 *Schistosoma mansoni*-cercaria 10 weeks previously were challenged with 1,500 SPZ of *P*. *yoelii* along with *S*. *mansoni-*non-infected controls. BALB/c: ***P<0.001, t = 6.283, df = 8; B6: *P<0.05, t = 2.511, df = 8; CBA: **P<0.01, t = 4.220, df = 7. (B) Copy number of *Plasmodium berghei* 18s RNA gene per 1×10^6^ mouse G3PDH gene measured at 42 h post sporozoite inoculation. Female BALB/c, B6, and CBA/J mice (N = 5) infected with 50 *S*. *mansoni*-cercaria 10 weeks previously were challenged with 1,500 SPZ of *P*. *berghei* along with *S*. *mansoni-*non-infected controls. BALB/c: *P<0.05, t = 2.306, df = 8; B6: ***P<0.001, t = 5.336, df = 8; CBA: *P<0.05, t = 2.846, df = 7. All data were statistically examined using Student’s two-tailed t-test.

Both B6 and CBA/J mice showed significant reduction in *P*. *yoelii* liver burden in *S*. *mansoni*-infected mice, consistent with the results observed in BALB/c mice (Fig 2A). The liver-stage growth of *P*. *berghei* was also reduced in *S*. *mansoni*-infected mice in all mouse strains examined (Fig 2B).

### *S*. *mansoni* infection significantly reduces liver-stage malaria parasite burden from 2 h following SPZ inoculation

**Fig 3:**
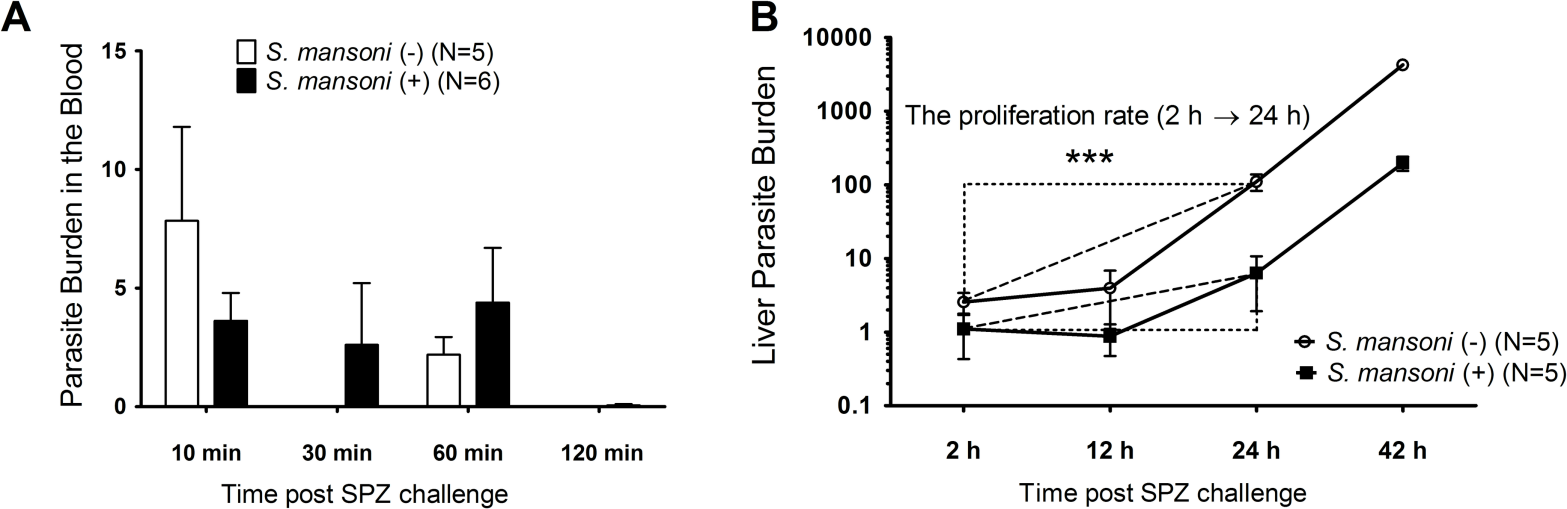
*Plasmodium yoelii* parasite density in the blood and liver. Copy number of *P*. *yoelii* 18s RNA gene per 1×10^6^ mouse G3PDH gene measured at 42 h post sporozoite inoculation. Female BALB/c mice (N = 5) infected with 50 *Schistosoma mansoni*-cercaria 10 weeks previously were challenged with 1,500 SPZ of *P*. *yoelii* along with *S*. *mansoni*-non-infected controls. (A) *P*. *yoelii* parasitaemia in the blood. (B) *P*. *yoelii* parasite density and proliferation in the liver. ***P<0.001, Student’s two-tailed t-test, t = -6.316, df = 8.

There are several potential mechanisms for the reduction in malaria parasite burden at 42 hours post SPZ inoculation in *S*. *mansoni* infected mice. It is possible, for example, that antibodies raised against schistosomes may offer protection against sporozoites through cross-reactivity [33]. It is also possible that liver fibrosis caused by schistosomiasis may physically impede the invasion of hepatocytes by sporozoites. Alternatively, there may be suppression of *P*. *yoelii* hepatocytic stages in the liver mediated by an altered immune environment in the liver caused by the presence *of S*. *mansoni* eggs in the organ. In order to investigate at which point *S*. *mansoni*-mediated reduction of malaria parasite in the liver occurs, we measured the number of malaria parasites in both the blood and the liver at time points throughout the 48 h growth of parasites in the liver. Ten microliters of blood were sampled from mouse tail-veins at 10, 30, 60, and 120 min, and livers were harvested at 2, 12, 24, and 42 h after i.v. inoculation of 1,500 SPZ. We found that malaria parasite load in the blood was not significantly different in infected compared with non-infected mice at any time point following SPZ inoculation (Fig 3A). In contrast, the number of malaria parasites in the liver were significantly reduced in *S*. *mansoni* infected mice from the first 2 h of development in the liver, with the proliferation rate from 2 to 24 h of liver stage parasites also suppressed in these mice (Fig 3B).

### Intraportal inoculation of frozen *S*. *mansoni-*eggs significantly reduces liver-stage malaria parasite burden

**Fig 4:**
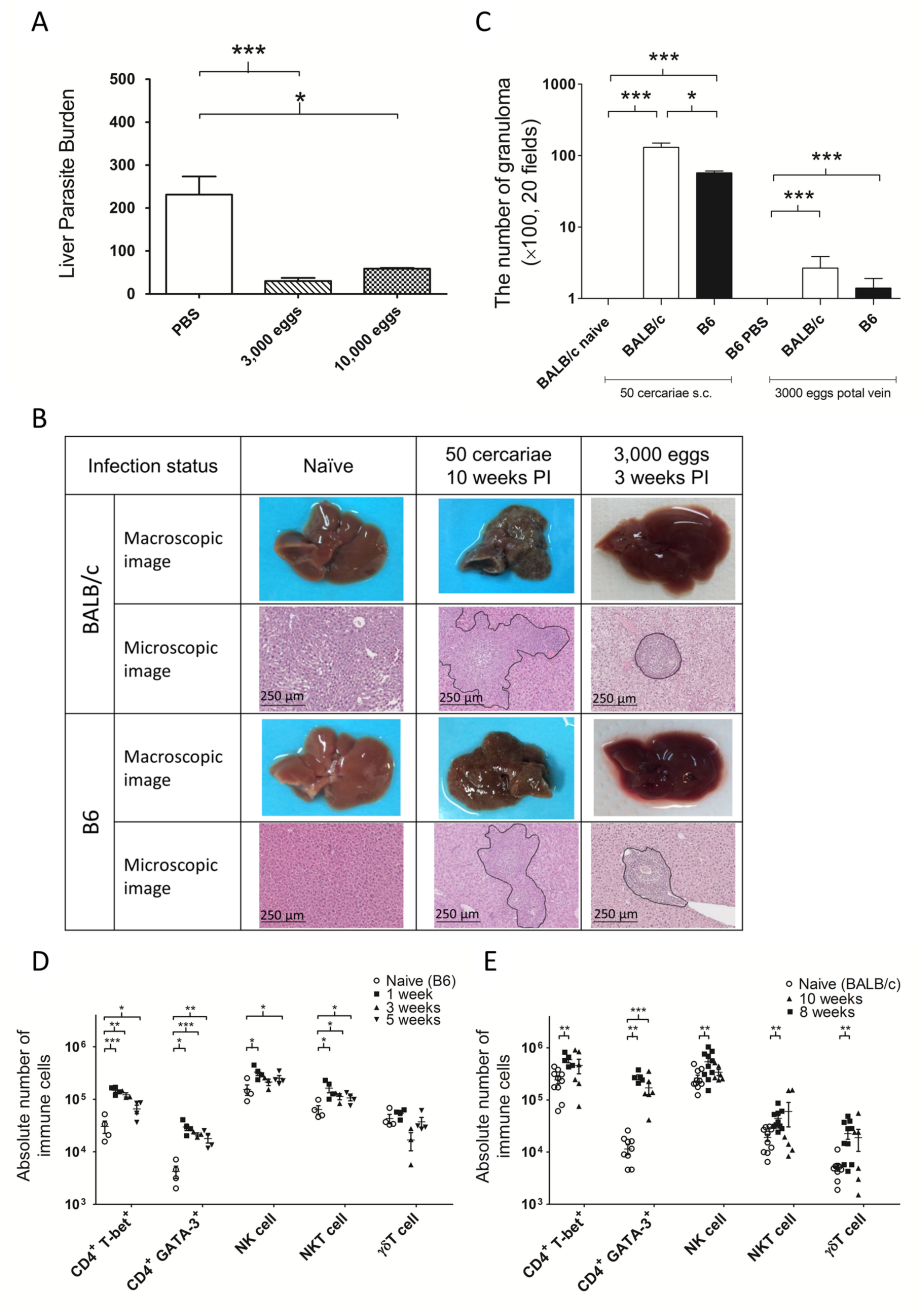
Liver immunopathology in *Schistosoma mansoni*-cercariae infection and intraportal infusion of frozen *S*. *mansoni*-eggs. (A) Malaria parasite liver burden with/without intraportal infusion of frozen *S*. *mansoni*-eggs. Female B6 mice were intraportally inoculated 3,000/10,000 frozen *S*. *mansoni*-eggs and challenged with 1,500 sporozoites of *Plasmodium yoelii* along with controls inoculated with PBS (control: N = 3; 3,000 frozen eggs: N = 5, ***P<0.001, t = 1.943, df = 6; 10,000 frozen eggs: N = 3, *P<0.05, Student’s two-tailed t-test, t = 4.072, df = 4). (B) Macroscopic and microscopic images of liver pathology. The black lines indicate 250 micro meters. (C) The number of granulomas in the liver. Female BALB/c mice and B6 mice were inoculated with 50 *S*. *mansoni*-cercariae subcutaneously (naive: N = 5; 50 cercariae: N = 3) or inoculated with 3,000 frozen *S*. *mansoni*-eggs along with controls inoculated with PBS intraportaly (PBS: N = 5; 3,000 frozen eggs: N = 5). The numbers of granuloma were counted in 20 microscopic fields at 100 x magnification. *P<0.05, ***P<0.001. All data were statistically examined using Student’s two-tailed t-test. (D) The numbers of immune cells induced by intraportal infusion of 3,000 frozen *S*. *mansoni*-eggs. Female B6 mice (N = 4/group) were intraportaly inoculated 3,000 *S*. *mansoni*-eggs. (E) The numbers of immune cells induced by 50 *S*. *mansoni*-cercaria infection. Female BALB/c mice (naive: N = 9, 8 weeks: N = 9, 10 weeks N = 6) were infected with 50 *S*. *mansoni*-cercariae. *P<0.05, **P<0.01, ***P<0.001. All data were statistically examined using Student’s two-tailed t-test.

As eggs are the principle cause of immunopathology in schistosomiasis [34], we assessed whether direct inoculation of *S*. *mansoni* eggs to the portal vein of mice could also reduce liver-stage malaria parasite growth. Female B6 mice were inoculated with 3,000 or 10,000 frozen *S*. *mansoni*-eggs in a total volume of 100 μL PBS into the portal vein. Control mice were inoculated with 100 μL PBS. Three weeks after egg inoculation, each group was challenged with 1,500 SPZ of *P*. *yoelii*. Liver-stage malaria parasite burden was significantly reduced in both groups inoculated with *S*. *mansoni*-eggs (Fig 4A).

*S*. *mansoni* infection causes severe liver damage associated with eosinophilic granulomas, collagen deposition, and fibrosis due to immunologic reactions to *Schistosoma* eggs trapped in the tissues. However, livers harvested from the intraportal infusion group did not show conspicuous damage macroscopically. Correspondingly, livers inoculated with *Schistosoma* eggs developed less and smaller granulomas compared with those harvested from the *S*. *mansoni*-cercariae infection group (Fig 4B and 4C).

*Schistosoma* infection is known to induce Th2-biased immune responses in mice. However, egg inoculation induced the infiltration of various immune cells into the liver with little pathology. Interferon-mediated innate immune responses are important modulators of malaria parasite growth in the liver [35]. We therefore focused not only on CD4^+^ T-bet^+^ cells and CD4^+^ GATA-3^+^ cells but also NK, NKT, and γδT cells which have previously been implicated as important sources of interferon-gamma (IFN-γ) in the liver during *Plasmodium* infection. The representative FACS plots are shown in the supplementary graph (S1 Fig). As shown in Fig 4D and 4E, the upregulation of the immune reaction induced by *S*. *mansoni* eggs in B6 mice was similar to that induced by *S*. *mansoni*-cercariae infection in BALB/c mice. Both CD4^+^ T-bet^+^ cells and CD4^+^ GATA-3^+^ cells significantly increased following intraportal inoculation of *S*. *mansoni*-eggs as well as after *S*. *mansoni*-cercariae infection. In both cases, the increase of CD4^+^ GATA-3^+^ cells was the largest among immune cells. The numbers of NK cells significantly increased only during the early phase, one week after intraportal inoculation of *S*. *mansoni*-eggs or 8 weeks after *S*. *mansoni*-cercariae infection. The numbers of NKT cells also significantly increased in both cases and this increase was greater in the group inoculated with *S*. *mansoni*-eggs. There was no significant increase in the numbers of γδT cells in either group. B6 mice infected with *S*. *mansoni*-cercariae showed a similar pattern of upregulation of immune cells (S2 Fig).

### The reduction of liver-stage malaria parasite burden was reversed in IFN-γ-deficient and IL-4-deficient mice

We hypothesised that the reduction of liver-stage malaria parasite burden is dependent on the immune environment such as IFN-γ production from host immune cells accumulated and activated by the presence *S*. *mansoni*-eggs in the liver. IFN-γ is a key cytokine for the control of malaria parasites and is induced in the liver during *S*. *mansoni* infection. Interleukin-4 (IL-4) is a major mediator for the induction of the immune response against *S*. *mansoni*. Therefore, we examined the impact of IFN-γ and IL-4 on liver-stage malaria parasite burden using IFN-γ-deficient (IFN-γ^-/-^) and IL-4-deficient (IL-4^-/-^) mice.

**Fig 5:**
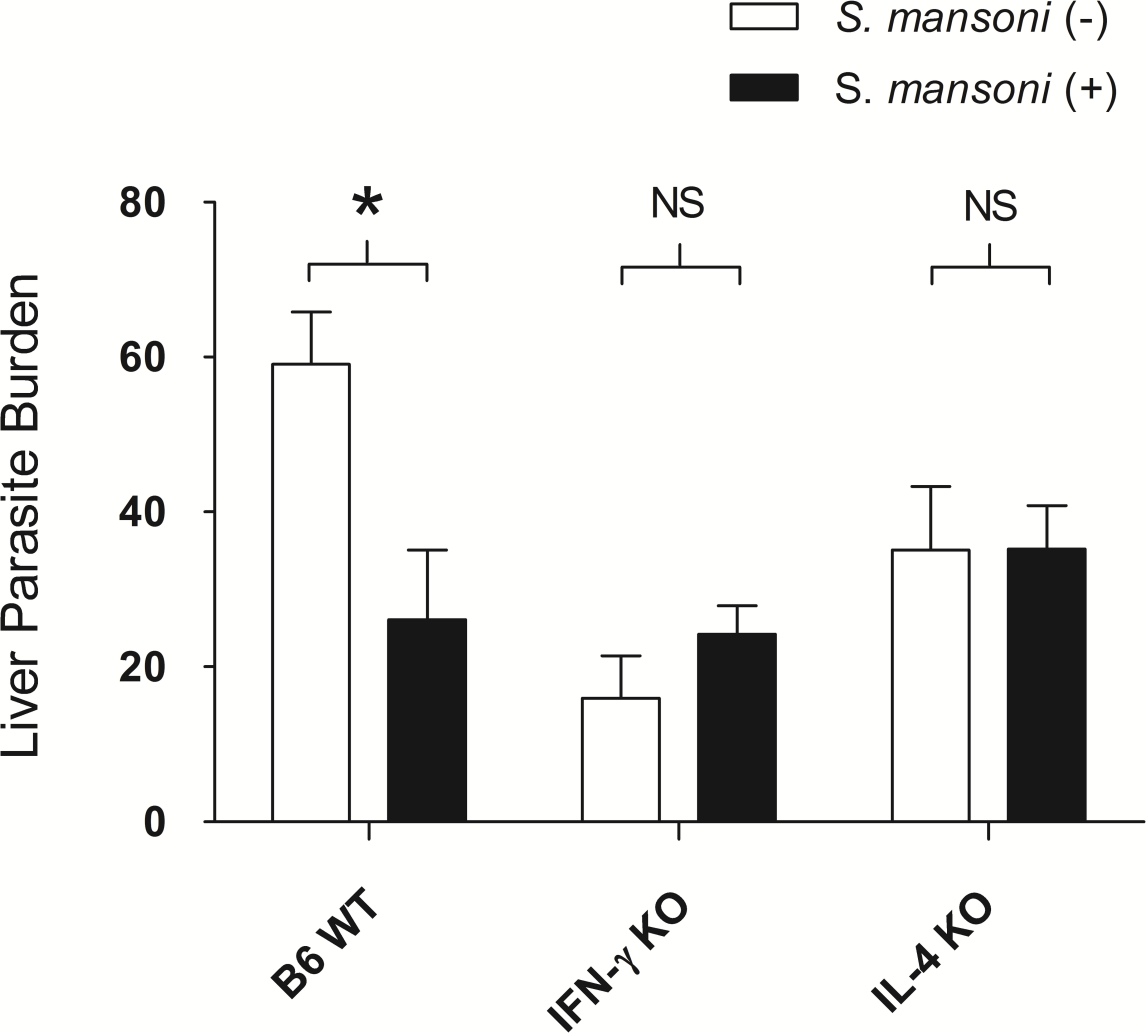
Malaria parasite liver burden in wild-type B6, IFN-gamma-deficient, and Interleukin-4-deficient mice. Copy number of *Plasmodium yoelii* 18s RNA gene per 1×10^6^ mouse G3PDH gene measured at 42 h post sporozoite inoculation. Female IFN-γ^-/-^ mice (N = 8), IL-4^-/-^ mice (N = 8), and B6 WT mice (N = 4) were intraportally inoculated with 3,000 frozen *S*. *mansoni*-eggs and challenged with 1,500 SPZ of *P*. *yoelii* along with each control groups inoculated 100 μL PBS (N = 4). B6 WT mice: *P<0.05, t = 3.017, df = 5; IFN-γ^-/-^ mice: Not significant (NS), t = -1.303, df = 10; IL-4-/- mice: NS, t = -0.016, df = 10.

IFN-γ^-/-^ and IL-4^-/-^ mice along with wild-type B6 (B6 WT) mice were inoculated 3,000 *S*. *mansoni*-eggs 3 weeks prior to SPZ challenge. Liver-stage malaria parasite burden was measured 42 h after i.v. challenge with 1,500 *P*. *yoelii* SPZ. Consistent with the result shown in Fig 4A, malaria liver burden was significantly reduced in the intraportal infusion group; however, this reduction was abrogated in IFN-γ^-/-^ and IL-4^-/-^ mice (Fig 5).

### *S*. *mansoni* infection inhibits the development of blood stage malaria following i.v. inoculation of entomologically relevant numbers (50) of SPZ

Through challenge with 1,500 SPZ of *P*. *yoelii*, we have demonstrated the impact of *S*. *mansoni* infection on liver-stage malaria parasite burden; this number of sporozoites, however, would not be expected to be inoculated naturally during a mosquito bite. In order to mimic the numbers inoculated by a mosquito bite, we reduced the challenge dose from 1,500 to 50 SPZ and examined the outcome of *Plasmodium* infection.

**Fig 6:**
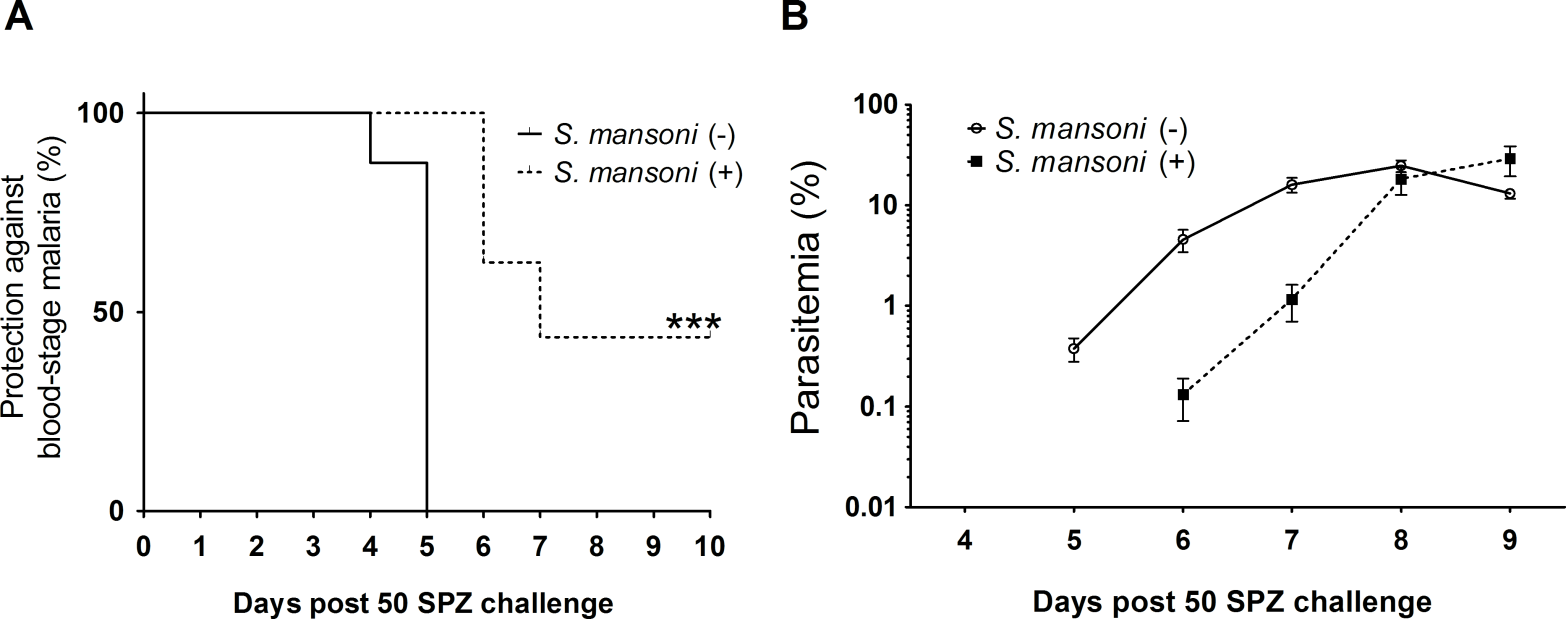
Malaria outcomes with low dose SPZ challenge. Female BALB/c mice (N = 16) infected with 50 *Schistosoma mansoni*-cercariae 10 weeks previously were challenged with 50 sporozoites of *Plasmodium yoelii* along with *S*. *mansoni*-non-infected controls. (A) The percentage of mice that did not develop blood stage infection following inoculation of low does SPZ. Data were statistically examined using the log-rank test. ***P<0.001, x2 = 29.8, df = 1. (B) Parasitaemia. Mean parasitaemia in *S*. *mansoni* infected group was calculated only among blood stage malaria positive mice.

BALB/c mice (N = 16) infected with 50 *S*. *mansoni*-cercariae 10 weeks previously along with non-infected controls were challenged with 50 *P*. *yoelii* SPZ. Seven out of 16 *S*. *mansoni*-coinfected mice did not develop malaria parasitaemia, in contrast to all non-*S*. *mansoni* infected control mice developing blood-stage malaria on day 5 after SPZ challenge (Fig 6A). When blood stage malaria parasite infection became patent in coinfected mice, it increased rapidly and matched the peak parasitaemia of non-*S*. *mansoni* infected controls (Fig 6B).

### *Plasmodium yoelii* infections in mice infected with *S*. *mansoni* were less infectious to mosquitoes

We measured the influence of *S*. *mansoni* on the infectivity of malaria parasites to mosquitoes. We allowed *Anopheles stephensi* mosquitoes to feed on mice infected with *S*. *mansoni* + *P*. *yoelii* or *P*. *yoelii* alone on days 3 and 4 post-infection, when *P*. *yoelii* is at its most infectious to mosquitoes. A minimum of eight mosquitoes were allowed to feed on each of 5 mice per group on each day, and the resulting infectivity measured by assessing the proportion of mosquitoes that were infected with oocysts eight days post-feeding, and through quantifying the numbers of oocysts per infected mosquito per group on each day of feeding. Prior to mosquito feeding, the parasitaemia and number of gametocytes circulating in the peripheral blood of mice was calculated via microscopy of thin blood films following i.v. inoculation of *P*. *yoelii*-parasitized erythrocytes.

**Fig 7:**
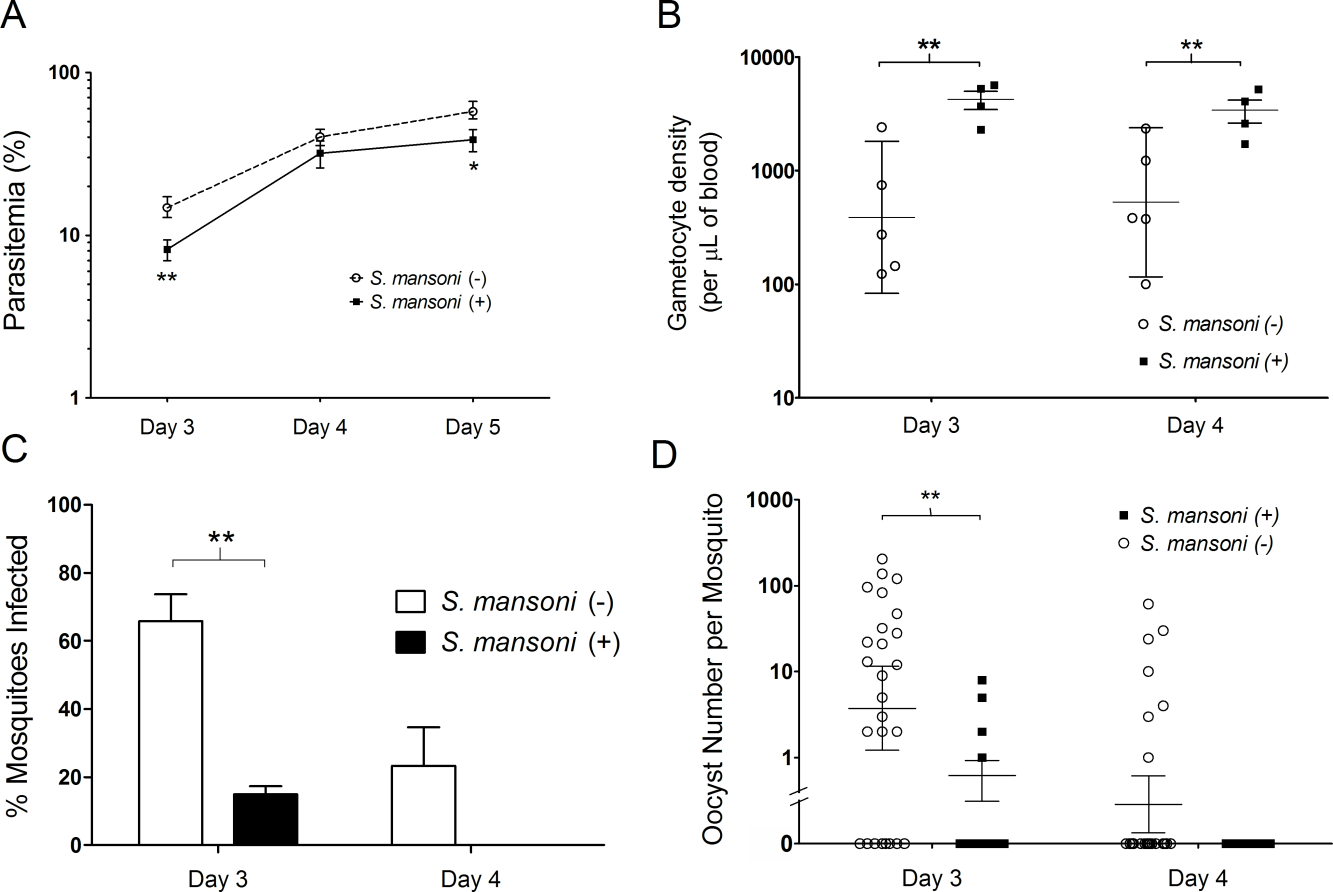
Gametocyte infectivity. (A) Parasitemia. Female BALB/c mice were each inoculated with 1 x 10^6^ *Plasmodium yoelii* parasitized erythrocytes intravenously with (n = 4 mice) or without (n = 5) pre-existing *Schistosoma mansoni* infection. Parasitaemia was determined by microscopic examination on days 3, 4, and 5 post-inoculation; day 3: Student’s two-tailed t-test; **P<0.01, t = -4.906, df = 7; Day5: *P<0.05, t = -2.922, df = 5 (**B**) Gametocyte density. Gametocyte density was determined on days 3 and 4 post-inoculation of 1 x 10^6^ *P*. *yoelii*-parasitized erythrocytes intravenously. Day 3: Student’s two-tailed t-test; **P<0.01, t = 3.813, df = 5; Day 4: **P<0.01, t = 3.608, df = 5. Error bars show the geometric mean with 95% confidence intervals. (C) Percentage of mosquitoes with one or more oocysts present on the midgut eight days post-feeding on infected mice. A minimum of eight mosquitoes were allowed to feed on each individual mouse in the group per day **P = 0.0003, (2-way ANOVA, F = 22.23, DFn = 1, DFd = 14). Error bars mar the standard error of the mean per mouse group. (D) Oocyst numbers per mosquito. The numbers of oocysts present on mosquito midguts were determined eight days post-mosquito feeding; day 3: Student’s two-tailed t-test, **P<0.01, t = 3.077, df = 25. Error bars show the geometric mean with 95% confidence intervals. Data is representative of three independent experiments.

The number of gametocytes circulating in *S*. *mansoni*-infected mice was significantly higher than that of mice without *S*. *mansoni* infection on days 3 and 4 (Fig 7B). Despite this, the proportion of mosquitoes infected with oocysts eight days post blood feed was significantly higher for the *S*. *mansoni* uninfected mic, compared to mice infected with *S*. *mansoni* (Fig 7C). Furthermore, oocyst numbers were significantly reduced in mosquitoes that had fed on mice infected *with S*. *mansoni*, compared to uninfected controls (Fig 7D).

## Discussion

We have examined the impact of schistosome infection on malaria pathology and transmission capacity in a mouse model. Previous animal studies on the interactions between *Plasmodium* and *Schistosoma* have focused exclusively on the blood stage pathology of the malaria parasite infection, and have consistently shown that parasitaemia is enhanced in the presence of the helminth [10–13]. We have investigated the effect of helminth-malaria parasite coinfection on the growth of rodent malaria parasites in the liver, and show that *S*. *mansoni*-coinfection significantly reduces *Plasmodium* parasite numbers in the liver following sporozoite inoculation. *S*. *mansoni*-coinfection with malaria parasites resulted in a large reduction in the percentage of mice developing blood stage malaria parasite infection when mice were challenged with numbers of SPZ similar to those inoculated during natural inoculation through the bites of infected mosquitoes [36–39].

These results might suggest a possible explanation for the often contradictory results observed in epidemiological studies. Some prospective cohort studies in human populations have suggested a protective effect of *Schistosoma* infection on *Plasmodium* infection: Lyke et al. concluded that children infected with *Schistosoma haematobium* showed increased time to first clinical malaria infection and fewer malaria episodes over the follow-up period compared to children without *S*. *haematobium* infection [7]. Doumbo et al. also showed that people coinfected with *S*. *haematobium* showed significant delays in time-to-first malaria episode[9]. Hürlimann et al. demonstrated that the *Plasmodium* parasitaemia incidence rate of children infected with *S*. *mansoni* increased after treatment with praziquantel; in contrast, children infected with hookworms had reduced *Plasmodium* parasitaemia rates following albendazole treatment [40]. Our results, which show that *S*. *mansoni* infection reduces the malaria parasite burden in the liver and the number of hosts developed blood stage infection following SPZ inoculation, may account for some of the observed protective effects of *Schistosoma* infection against malaria.

The intraportal inoculation of *S*. *mansoni* eggs reduced malaria parasite burden in the liver to the same extent as inoculation of cercariae. As shown in Fig 4, intraportal inoculation of *S*. *mansoni*-eggs induced an increase in the numbers of various lymphocytes, including both Th1 and Th2 cells, NK, NKT, and γδT cells in the liver without apparent fibrotic or granulomatous liver damage. This suggests that the suppression of the growth of malaria parasites in the liver is not mediated by physiological or morphological changes of the liver environment provoked by *S*. *mansoni*-eggs, but rather by changes in the hepatic immune microenvironment.

Previous animal studies have shown that interferon-mediated innate immune responses are important modulators of malaria parasite growth in the liver [41]; Miller et al. have shown that IFN-γ production from NKT cells (but not NK cells or T cells) is critical in reducing malaria parasite burden in the liver [35]. We initially hypothesised that malaria parasite liver burden would increase in *S*. *mansoni*-coinfected mice as *S*. *mansoni* infection has been shown to induce robust Th2 type immune responses with a corresponding downregulation of Th1 responses [42]. However, contrary to our expectations, malaria parasite liver burden was significantly reduced in *S*. *mansoni*-coinfected mice. Both IFN-γ and IL-4 are required for this effect, and both the inoculation of *S*. *mansoni* eggs to the hepatic portal vein, and the infection of mice with cercariae were shown to increase innate immune cells and both Th1 and Th2 cells in the liver (S2 Fig). Taken together, this suggest that possible sources of IFN-γ and IL-4 include not only innate immune cells such as NKT cells but also helper T cells accumulated and activated by *S*. *mansoni* infection in the liver [43].

IFN-γ^-/-^ and IL-4^-/-^ mice had lower numbers of malaria parasites in the liver after SPZ challenge compared with B6 WT mice even without *S*. *mansoni* infection. The experiment was repeated twice with similar results. These results are in agreement with previous studies with IL-4^-/-^ mice although the mechanisms are not fully understood. IL-4^-/-^ mice are known to be more resistant to sporozoite infection than wild-type mice owing to increased NK cell numbers and expression of inducible nitric oxide synthase in the liver [44]. Since the reduction of malaria parasite burden in the liver in co-infected mice was reversed in IFN-γ^-/-^ and IL-4^-/-^ mice, we assume that the mechanism of protection is both IFN-γ and IL-4 dependent. To elucidate the mechanisms underlying this phenomenon, further research into the interactions between *Plasmodium* and *Schistosoma* are required.

We demonstrated that the infectivity of *Plasmodium* gametocytes to *Anopheles* mosquitoes dropped abruptly from a peak on day 3 to zero on day 4 in control mice not infected with *S*. *mansoni*. This phenomenon is known in animal models as malaria infection crisis and has previously been reported to occur in numerous *Plasmodium* species [45]. The mechanisms behind this loss of infectivity have not been fully explained, but previous animal studies suggest that cytokines such as tumor necrosis factor (TNF), IFN-γ, interleukin-6 (IL-6), and nitric oxide may inhibit the development of gametocytes [25–27]. The infectivity of *P*. *yoelii* gametocytes in *S*. *mansoni* infected mice was significantly reduced on day 3 post-inoculation which is the peak of infectivity in non-*S*. *mansoni* infected mice. Observing higher levels of IFN-γ in the sera of *S*. *mansoni* infected mice on day 3 and day 4 (S3 Fig), we assume that pre-existing *S*. *mansoni* infection induces activation of the host immune environment and this leads to an accelerated infection crisis. Although the mechanisms remain to be elucidated, this experimental transmission model suggests that *S*. *mansoni* infection may reduce malaria transmissibility from mosquitoes to mice.

In conclusion, the presence of *S*. *mansoni* infection dramatically reduced the number of rodent malaria parasites in the liver. This reduction leads to the inhibition of the development of blood stage malaria parasite infection following inoculation of biologically relevant numbers of sporozoites. We also demonstrate that *P*. *yoelii* gametocyte infectivity to mosquitoes is significantly reduced in the presence of *S*. *mansoni* in co-infected mice. These results imply that *S*. *mansoni* infection can reduce malaria transmission both from mosquitoes to mice and from mice to mosquitoes, and may explain some of the protective effects of *Schistosoma* infection against malaria described in previous epidemiological studies.

## Supporting information

S1 Fig Representative flow cytometry data of immune cells in the liver. Hepatic nonparenchymal cells were isolated from C57BL/6 mice at 1, 3 and 5 weeks post 3000 *S*. *mansoni* frozen eggs portal vein inoculation, or from BALB/c mice at 8 and10 weeks post 50 *S*. *mansoni*-cercariae s.c. inoculation.
(PDF)

S2 Fig The numbers of immune cells induced by 50 *Schistosoma mansoni*-cercariae infection. Female B6 mice (N = 5/group) were infected with 50 *S*. *mansoni*-cercariae. *P<0.05, **P<0.01, ***P<0.001, Student’s two-tailed t-test.
(PDF)

S3 Fig Concentration of IFN-γ in the serum. Female BALB/c mice were inoculated one million *Plasmodium yoelii*-parasitized erythrocytes intravenously with or without pre-existing *Schistosoma mansoni* infection. On day 3 and 4 after inoculation, the concentration of interferon-gamma (IFN-γ) was measured in the serum. Day 3:*P<0.05, t = -2.209, df = 6; Day 4:*P<0.05, t = -2.516, df = 4, Student’s two-tailed t-test.
(PDF)
