# Supplementary material for: Schistosoma mansoni infection suppresses the growth of Plasmodium yoelii parasites in the liver and reduces gametocyte infectivity to mosquitoes
Source: PLoS Negl Trop Dis. 2018 Jan 26;12(1):e0006197. doi: 10.1371/journal.pntd.0006197 (PMC5802944; doi:10.1371/journal.pntd.0006197)
Supplement: S1 Fig — Hepatic nonparenchymal cells were isolated from C57BL/6 mice at 1, 3 and 5 weeks post 3000 S. mansoni frozen eggs portal vein inoculation, or from BALB/c mice at 8 and10 weeks post 50 S. mansoni-cercariae s.c. inoculation. (PDF) [file pntd.0006197.s001.pdf]

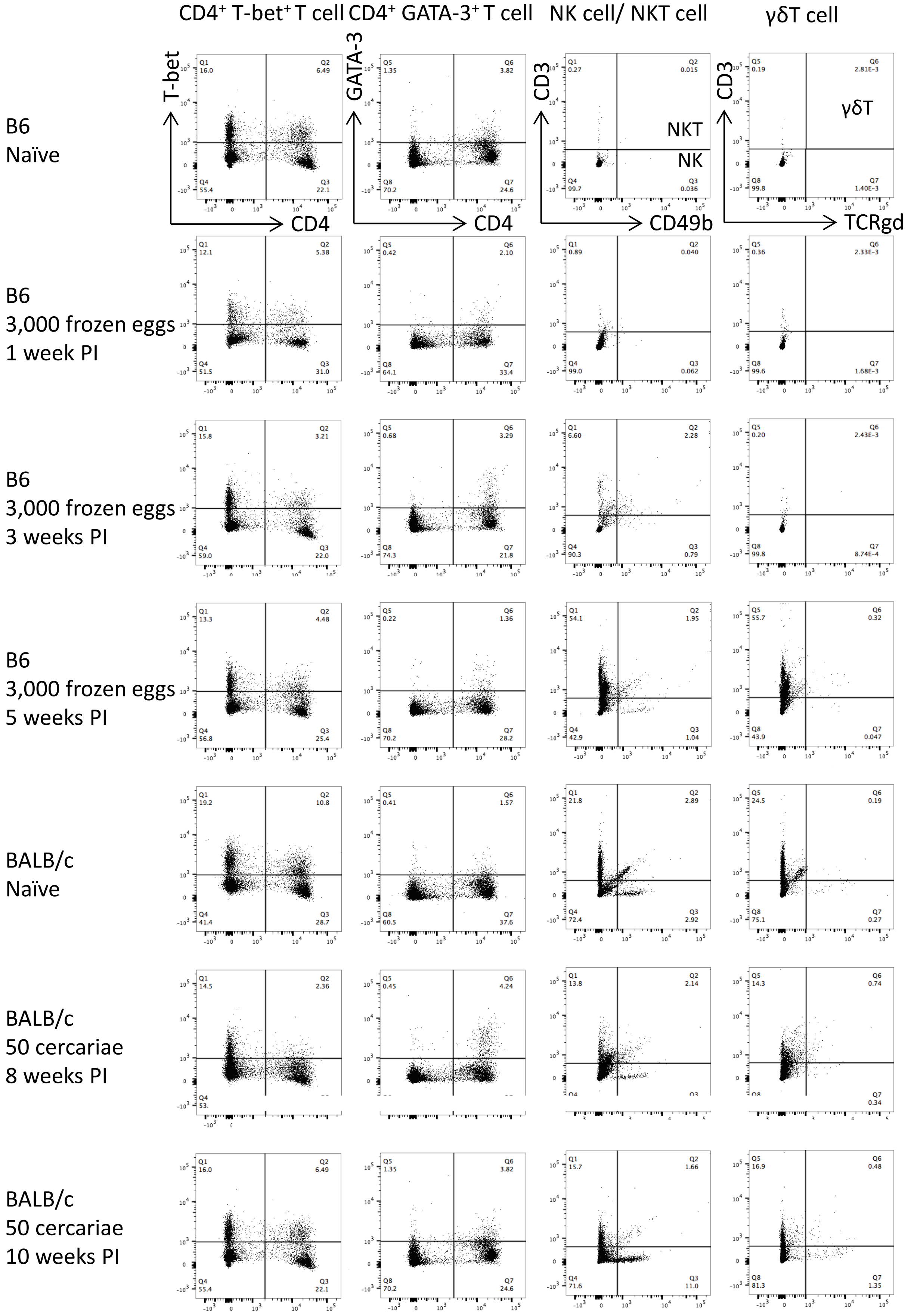
CD4⁺ T-bet⁺ T cell
CD4⁺ GATA-3⁺ T cell
NK cell/ NKT cell
γδT cell
T-bet
GATA-3
CD3
CD3
CD4
CD4
CD49b
TCRgd
NKT
NK
γδT
B6
Naïve
B6
3,000 frozen eggs
1 week PI
B6
3,000 frozen eggs
3 weeks PI
B6
3,000 frozen eggs
5 weeks PI
BALB/c
Naïve
BALB/c
50 cercariae
8 weeks PI
BALB/c
50 cercariae
10 weeks PI
