# Supplementary figures and images for: Schistosoma mansoni infection suppresses the growth of Plasmodium yoelii parasites in the liver and reduces gametocyte infectivity to mosquitoes
Source: PLoS Negl Trop Dis. 2018 Jan 26;12(1):e0006197. doi: 10.1371/journal.pntd.0006197 (PMC5802944; doi:10.1371/journal.pntd.0006197)

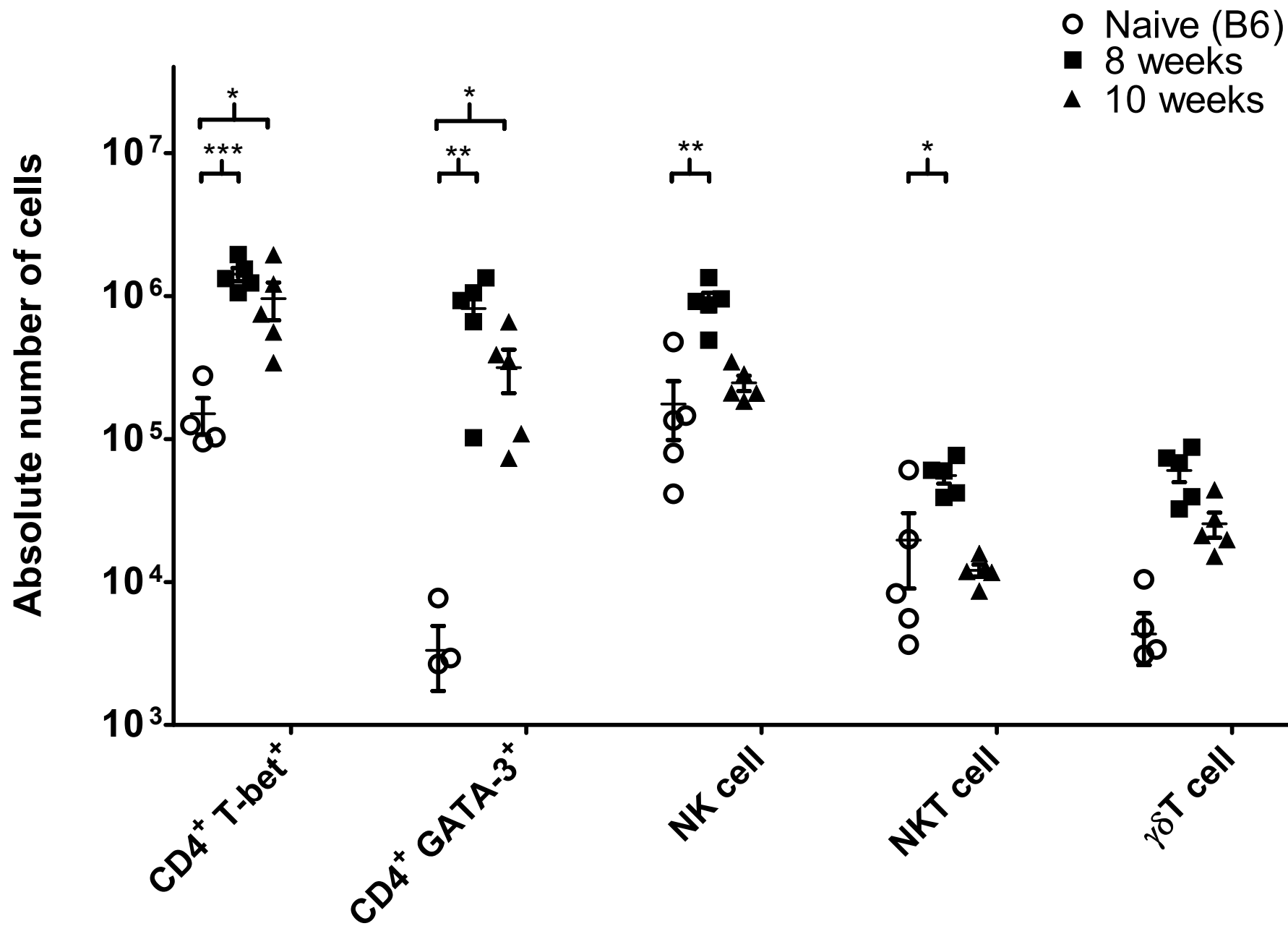

Supplement: S2 Fig — Female B6 mice (N = 5/group) were infected with 50 S. mansoni-cercariae. *P<0.05, **P<0.01, ***P<0.001, Student’s two-tailed t-test. (PDF) [file pntd.0006197.s002.pdf]

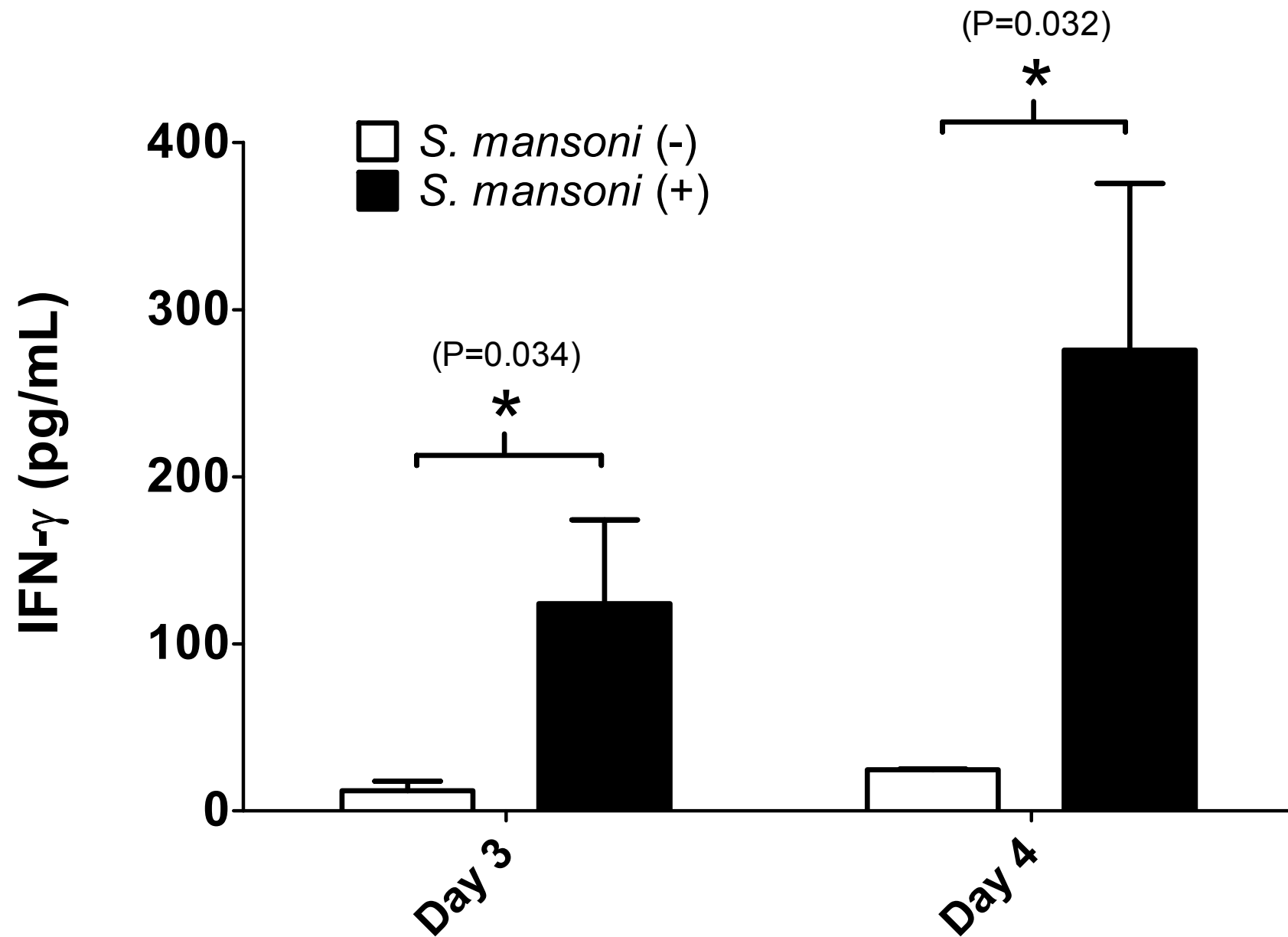

Supplement: S3 Fig — Female BALB/c mice were inoculated one million Plasmodium yoelii-parasitized erythrocytes intravenously with or without pre-existing Schistosoma mansoni infection. On day 3 and 4 after inoculation, the concentration of interferon-gamma (IFN-γ) was measured in the serum. Day 3:*P<0.05, t = -2.209, df = 6; Day 4:*P<0.05, t = -2.516, df = 4, Student’s two-tailed t-test. (PDF) [file pntd.0006197.s003.pdf]
